# Anti-S2 antibodies responsible for the SARS-CoV-2 infection-induced serological cross-reactivity against MERS-CoV and MERS-related coronaviruses

**DOI:** 10.3389/fimmu.2025.1541269

**Published:** 2025-03-28

**Authors:** Siyuan Sun, Jiaying He, Luotian Liu, Yuzhen Zhu, Qingsong Zhang, Yinong Qiu, Yuru Han, Song Xue, Xiaofang Peng, Yiming Long, Tianyu Lu, Wei Wu, Anqi Xia, Yunjiao Zhou, Yan Yan, Yidan Gao, Lu Lu, Lei Sun, Minxiang Xie, Qiao Wang

**Affiliations:** ^1^ Key Laboratory of Medical Molecular Virology (MOE/NHC/CAMS), Shanghai Institute of Infectious Disease and Biosecurity, Shanghai Frontiers Science Center of Pathogenic Microorganisms and Infection, Shanghai Fifth People’s Hospital, Shanghai Key Laboratory of Medical Epigenetics, Institutes of Biomedical Sciences, School of Basic Medical Sciences, Fudan University, Shanghai, China; ^2^ Microbiological Testing Department, Baoshan District Center for Disease Control and Prevention, Shanghai, China; ^3^ Department of Gastroenterology, Jingan District Central Hospitals, Fudan University, Shanghai, China; ^4^ Fundamental Research Center, Shanghai Yangzhi Rehabilitation Hospital (Shanghai Sunshine Rehabilitation Center), School of Medicine, Tongji University, Shanghai, China

**Keywords:** SARS-CoV-2, MERS-CoV, MERS-related coronavirus, cross-reactivity, neutralization, S2 domain

## Abstract

Sarbecoviruses, such as SARS-CoV-2, utilize angiotensin-converting enzyme 2 (ACE2) as the entry receptor; while merbecoviruses, such as MERS-CoV, use dipeptidyl peptidase 4 (DPP4) for viral entry. Recently, several MERS-related coronaviruses, NeoCoV and PDF-2180, were reported to use ACE2, the same receptor as SARS-CoV-2, to enter cells, raising the possibility of potential recombination between SARS-CoV-2 and MERS-related coronaviruses within the co-infected ACE2-expressing cells. However, facing this potential recombination risk, the serum and antibody cross-reactivity against MERS/MERS-related coronaviruses after SARS-CoV-2 vaccination and/or infection is still elusive. Here, in this study, we showed that the serological cross-reactivity against MERS/MERS-related S proteins could be induced by SARS-CoV-2 infection but not by inactivated SARS-CoV-2 vaccination. Further investigation revealed that this serum cross-reactivity is due to monoclonals recognizing relatively conserved S2 epitopes, such as fusion peptide and stem helix, but not by antibodies against the receptor-binding domain (RBD), N-terminal domain (NTD) or subdomain-1 (SD1). Some of these anti-S2 cross-reactive mAbs showed cross-neutralizing activity, while none of them exhibited antibody-dependent enhancement (ADE) effect of viral entry *in vitro*. Together, these results dissected the SARS-CoV-2 infection-induced serological cross-reactivity against MERS/MERS-related coronaviruses, and highlighted the significance of conserved S2 region for the design and development of pan-β-coronaviruses vaccines.

## Introduction

Coronaviruses are a large family and could be classified into four genera: α, β, γ and δ. The α and β coronaviruses usually infect mammals, such as bats and humans; while the γ and δ coronaviruses mainly infect birds and occasionally mammals (1). During the past two decades, three types of coronaviruses have caused three major outbreaks, including severe acute respiratory syndrome coronavirus (SARS-CoV) in November 2002, Middle East respiratory syndrome coronavirus (MERS-CoV) in April 2012, and SARS-CoV-2 in December 2019 (1–4). Interestingly, all these three types of coronaviruses belong to β-coronaviruses.

Since 2019, the COVID-19 outbreak and the subsequent emergence of a variety of SARS-CoV-2 variants has brought great damage to human health and social economy. SARS-CoV-2, as a type of positive-strand RNA virus, belongs to the coronavirus family. The spike (S) protein of SARS-CoV-2, as one of its structural proteins, mediates viral entry into host cells and is the main target for neutralizing antibodies (5). The S protein of SARS-CoV-2 exhibits high levels of genetic diversity due to the high error rate during viral replication (6). Consequently, novel amino acid substitutions emerged and are emerging on the S protein, contributing to the viral infection efficiency and immune evasion capacity for the variety of SARS-CoV-2 variants, such as Alpha, Delta, Omicron and so on. Moreover, importantly, frequent recombination among different SARS-CoV-2 variants triggered new types of recombinant sublineages. For example, XBB variant was originated from the recombination of two Omicron subvariants, BJ.1 and BM.1.1.1 (7). Most recently, a circulating variant called XEC is the product of a recombination between two JN.1-originated variants, KS.1.1 and KP.3.3 (8).

**Figure 1:**
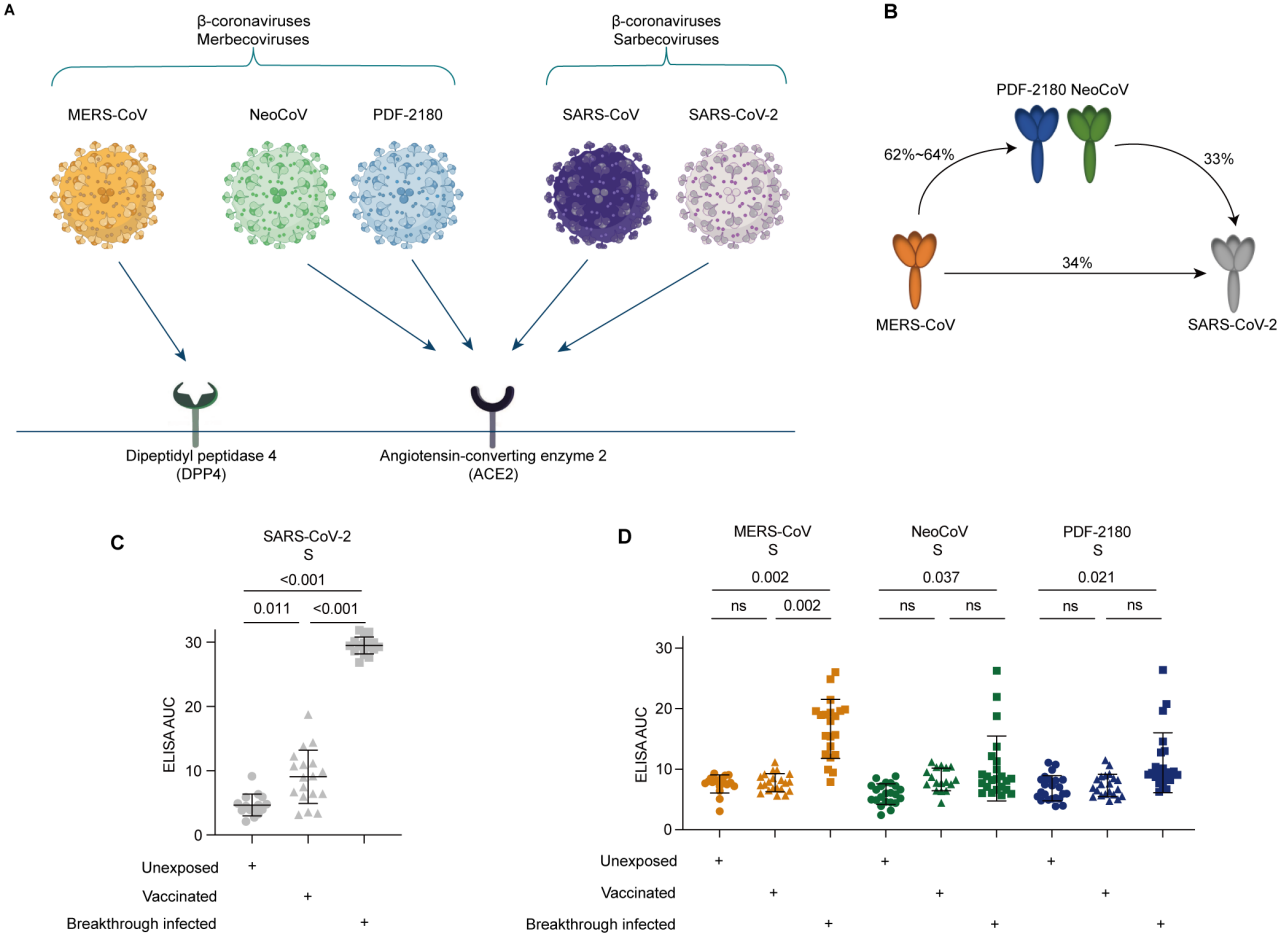
Serological cross-reactivity against the S proteins of MERS-CoV and its related NeoCoV and PDF-2180 coronaviruses. **(A)** Differential receptor usage for distinct β-coronaviruses. **(B)** Percentages of amino acid similarity among distinct S proteins. **(C)** Serological ELISA against the S protein of SARS-CoV-2. **(D)** Serological ELISA against the S proteins of MERS-CoV, NeoCoV, and PDF-2180. Statistical significance differences are determined by one-way ANOVA. Data are presented as the mean ± SD. ns, not significant.

Compared with SARS-CoV-2, MERS-CoV infection is more deadly, with mortality rate up to approximately 35%, more than 100 times higher than that of SARS-CoV-2 infection (9). Distinct from the angiotensin-converting enzyme 2 (ACE2) receptor used by SARS-CoV-2 for viral entry, MERS-CoV used receptor-binding domain (RBD) located in the S1 region to bind the dipeptidyl peptidase 4 (DPP4) receptor to initiate the viral entry (**Figure 1A**) (10, 11).

Surprisingly, two bat coronaviruses, NeoCoV and PDF-2180, which also belongs to β-coronaviruses but are closely related to MERS-CoV (62.3% and 63.5% amino acid sequence similarity with the S protein of MERS-CoV), have recently been reported to use ACE2, but not DPP4 as their viral entry receptor (**Figure 1A**) (10). These findings uncovered a potential threat that, during evolution, the MERS-related coronavirus might achieve the ability to use human ACE2 receptor to enter the host cells. Consequently, the co-infection of SARS-CoV-2 and MERS-related coronaviruses within the ACE2-expressing cells might lead to genetic recombination between these two different strains of coronaviruses. Through such potential recombination, a new clade of β-coronaviruses, such as SARS-CoV-3 or MERS-CoV-2, with an Omicron-like high transmissibility and a MERS-CoV-like high fatality rate, might be formed, posing a great threat to global public health and generating a catastrophic consequence of mortality (12).

Facing this potential threat, it is crucial and necessary to understand the antibody cross-reactivity against MERS/MERS-related coronaviruses after SARS-CoV-2 vaccination and/or infection. A recent study showed that vaccinated individuals with SARS-CoV-2 BA.5/BF.7 breakthrough infection induced no serological cross-reactivity against MERS-CoV and NeoCoV (13). However, in this study, the authors only tested the cross-reactivity against the RBD domain, but not against the N-terminal domain (NTD), subdomain-1 (SD1), and S2 domain (**Supplementary Figure S1A**). Moreover, although several monoclonal antibodies against highly conserved cold linear epitopes, such as fusion peptide (aa 814-838), stem helix region (aa 1141-1161), and subdomain-1 (SD1, aa 520-581) showed broad cross-reactivity against various coronaviruses and distinct SARS-CoV-2 variants (14), their cross-reactivity and cross-neutralization against MERS-CoV and MERS-related coronaviruses have not been very well characterized.

Here, in this study, we recruited SARS-CoV-2 convalescent and vaccinated donors, and investigated their serological cross-reactivity against MERS-CoV and MERS-related coronaviruses (NeoCoV and PDF-2180). We found that, BA.2 or BA.5 breakthrough infection, but not vaccination by inactivated SARS-CoV-2 vaccines, induced serum cross-binding activity against the S proteins of MERS-CoV and MERS-related coronaviruses. Further dissection suggested the serological cross-reactivity against MERS-CoV and MERS-related coronaviruses was likely due to the monoclonals against fusion peptide and stem helix, but not due to anti-RBD, anti-NTD, or anti-SD1 antibodies. Moreover, some cross-reactive monoclonals also exhibited varying degrees of cross-neutralizing activity against MERS-CoV, NeoCoV and PDF-2180, but without inducing any antibody-dependent enhancement (ADE) effect of viral entry against MERS/MERS-related coronaviruses.

## Results

### Serum cross-reactivity against S proteins of MERS/MERS-related coronaviruses

Facing the potential risk of recombination between SARS-CoV-2 variants and the ACE2-dependent MERS-related coronaviruses, we would like to investigate the serological cross-reactivity induced by COVID-19 vaccination or SARS-CoV-2 breakthrough infection against MERS-CoV and its two related ACE2-dependent coronaviruses, NeoCoV and PDF-2180 (**Figure 1A**). Sequence homology analysis showed low levels of amino acid sequence similarity, only 33-34%, between SARS-CoV-2 and MERS/MERS-related coronaviruses (**Figure 1B**).

To do this, we then performed serological ELISA against mammalian cell-expressed recombinant S proteins (**Supplementary Figure S1B**). As expected, our recruited donors who have received two or three doses of inactivated SARS-CoV-2 vaccines (p=0.011) and who experienced BA.5/BF.7 breakthrough infection (p<0.001) showed significantly higher level of ELISA reactivity against the S protein of SARS-CoV-2, compared with the unexposed (unvaccinated and uninfected) donors (**Figure 1C**).

However, for the SARS-CoV-2 vaccinated donors, no significant serological cross-reactivity against the S proteins of MERS-CoV, NeoCoV, and PDF-2180 was observed, similar to the unexposed donors (**Figure 1D**). On the contrary, compared with the unexposed donors, the individuals with BA.5/BF.7 breakthrough infection showed 1.6-2.2-fold increase of ELISA AUC against the S proteins of MERS-CoV (p=0.002), NeoCoV (p=0.037), and PDF-2180 (p=0.021). Together, these results suggested that SARS-CoV-2 breakthrough infection but not COVID-19 vaccination could induce serological antibody cross-reactivity against MERS-CoV and its related coronaviruses NeoCoV and PDF-2180.

### Cross-reactivity of anti-RBD antibodies

**Figure 2:**
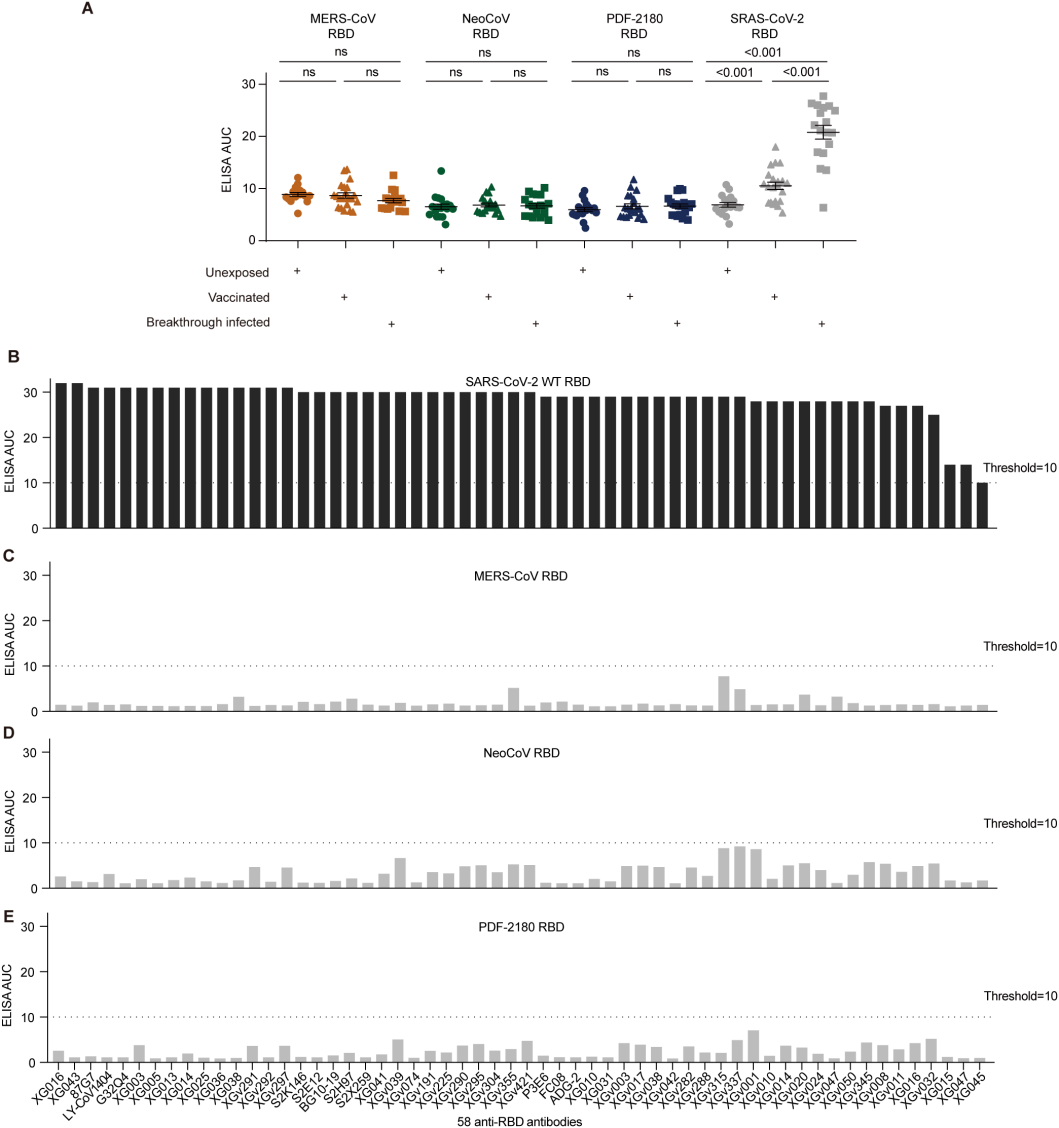
Antibody cross-reactivity for anti-RBD antibodies. **(A)** Serological ELISA against the RBD of MERS-CoV, NeoCoV, PDF-2180, and SARS-CoV-2. Statistical significance differences are determined by one-way ANOVA. **(B-E)** ELISA binding activity of a series of anti-SARS-CoV-2 RBD antibodies against the RBD proteins of SARS-CoV-2 **(B)**, MERS-CoV **(C)**, NeoCoV **(D)**, and PDF-2180 **(E)**. Representative of at least two independent experiments. ns, not significant.

We then would like to investigate the underlying mechanism of the serum cross-reactivity induced by SARS-CoV-2 breakthrough infection. Since the RBD of SARS-CoV-2 S protein has high immunogenicity and could induce robust antibody immune response (15–18), we first examined the serum cross-reactivity against the recombinant RBD proteins of MERS/MERS-related coronaviruses (**Supplementary Figure S1B**). As shown, although the vaccination and breakthrough infection induced significantly higher binding activity against SARS-CoV-2 RBD compared with the unexposed donors (p<0.001), no serological ELISA cross-reactivity was detected against the RBD proteins of MERS/MERS-related coronaviruses (**Figure 2A**).

We then used 58 anti-RBD monoclonal antibodies, including 15 XG series monoclonals identified from a convalescent donor (5), 32 XGv series monoclonals isolated from four vaccinated individuals (19), and 11 well-characterized broadly neutralizing antibodies, such as P3E6 (20), S2K146 (21), S2E12 (22), 87G7 (23), FC08 (24), BG10-19 (25), LY-CoV1404 (26), G32Q4 (27), ADG-2 (28), S2H97 (22), S2X259 (29). All these antibodies showed robust ELISA binding activity against the S protein of SARS-CoV-2 (**Figure 2B**). However, none of these tested antibodies showed robust cross-reactivity against the RBD proteins of MERS-CoV, NeoCoV, and PDF-2180, with their ELISA AUC below the threshold (**Figures 2C–E**). Therefore, we concluded that the serological cross-reactivity against the S proteins of MERS/MERS-related viruses was not due to the anti-RBD antibodies induced by vaccination and breakthrough infection.

### Cross-reactivity of anti-NTD and anti-SD1 antibodies

**Figure 3:**
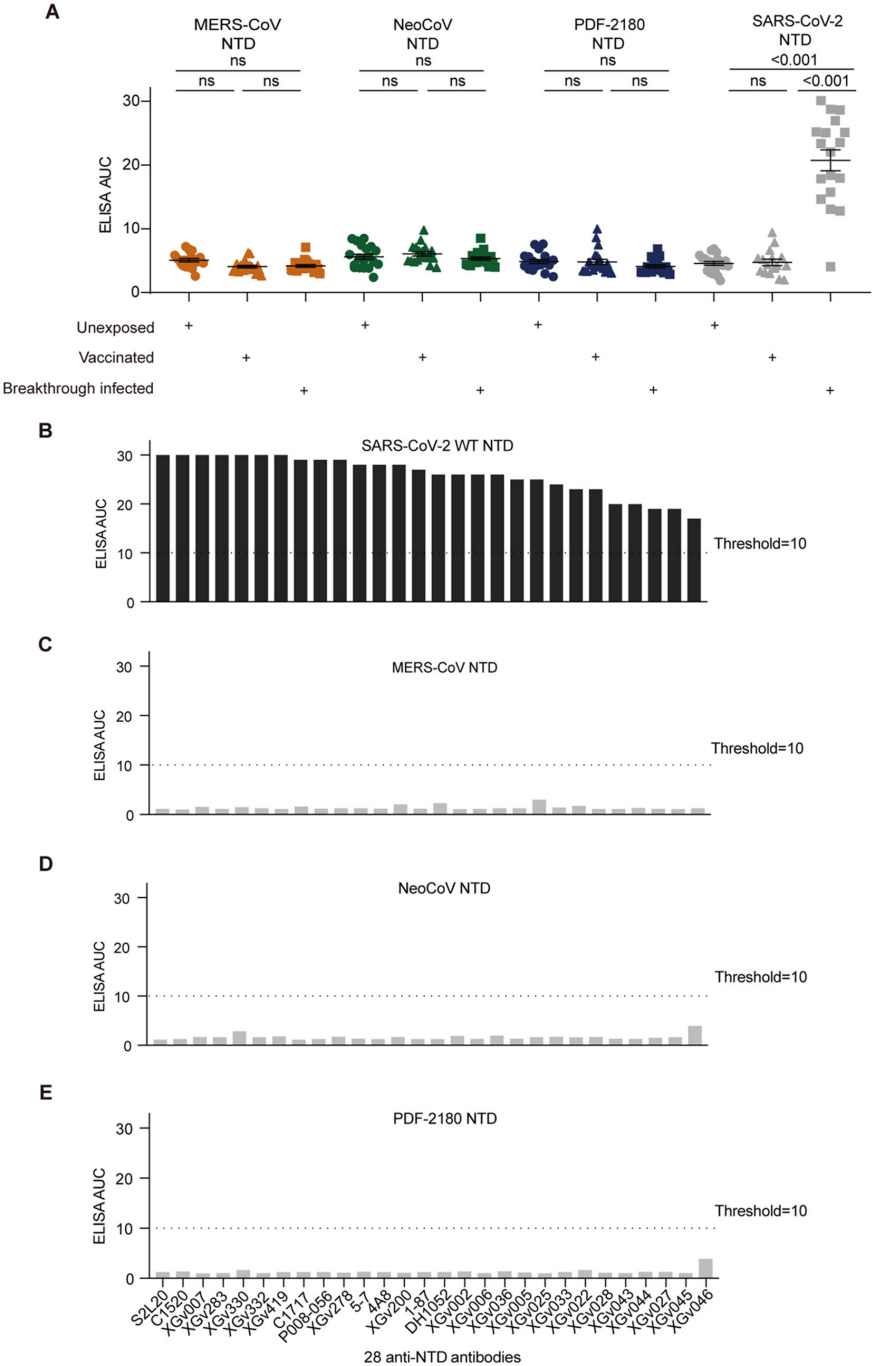
Antibody cross-reactivity for anti-NTD antibodies. **(A)** Serological ELISA against the NTD of MERS-CoV, NeoCoV, PDF-2180, and SARS-CoV-2. Statistical significance differences are determined by one-way ANOVA.**(B-E)** ELISA binding activity of a series of anti-SARS-CoV-2 NTD antibodies against the NTD proteins of SARS-CoV-2 **(B)**, MERS-CoV **(C)**, NeoCoV **(D)**, and PDF-2180 **(E)**. Representative of at least two independent experiments. ns, not significant.

SARS-CoV-2 infection also induced antibody response against the NTD of S protein (5, 15, 18). Similar to the ELISA results against RBD, the serological ELISA also showed that SARS-CoV-2 vaccination and breakthrough infection barely induced serum cross-reactivity against the NTD proteins of MERS-CoV, NeoCoV and PDF-2180 (**Figure 3A**). However, the serum cross-reactivity against SARS-CoV-2 NTD after breakthrough infection was significantly (p<0.001) higher than those of vaccinated and unexposed donors (**Figure 3A**).

We then selected 28 anti-NTD antibodies, including 20 XGv series monoclonals isolated from vaccinated donors (19) and eight previously-reported anti-NTD antibodies, such as 5-7 (18), C1717 (30), S2L20 (31), DH1052 (32), 1-87 (18), P008-056 (33), C1520 (30), and 4A8 (34). These antibodies showed efficient binding activity against the NTD protein of SARS-CoV-2 (**Figure 3B**), but no cross-binding activity against the NTD proteins of our tested three merbecoviruses (**Figures 3C–E**).

In addition, we further tested several XGv antibodies binding with S1 protein but not RBD/NTD of SARS-CoV-2 and an antibody sd1.040 targeting a conserved epitope on SD1 (14). None of these antibodies showed cross-reactivity against recombinant S proteins of MERS-CoV, NeoCoV, and PDF-2180 (**Supplementary Figures S2A**-**S2C**). Therefore, we concluded that the SARS-CoV-2 infection-induced serological cross-reactivity against MERS/MERS-related viruses was not caused by anti-S1 antibodies.

### Sequence similarity analysis of various domains on S protein

**Figure 4:**
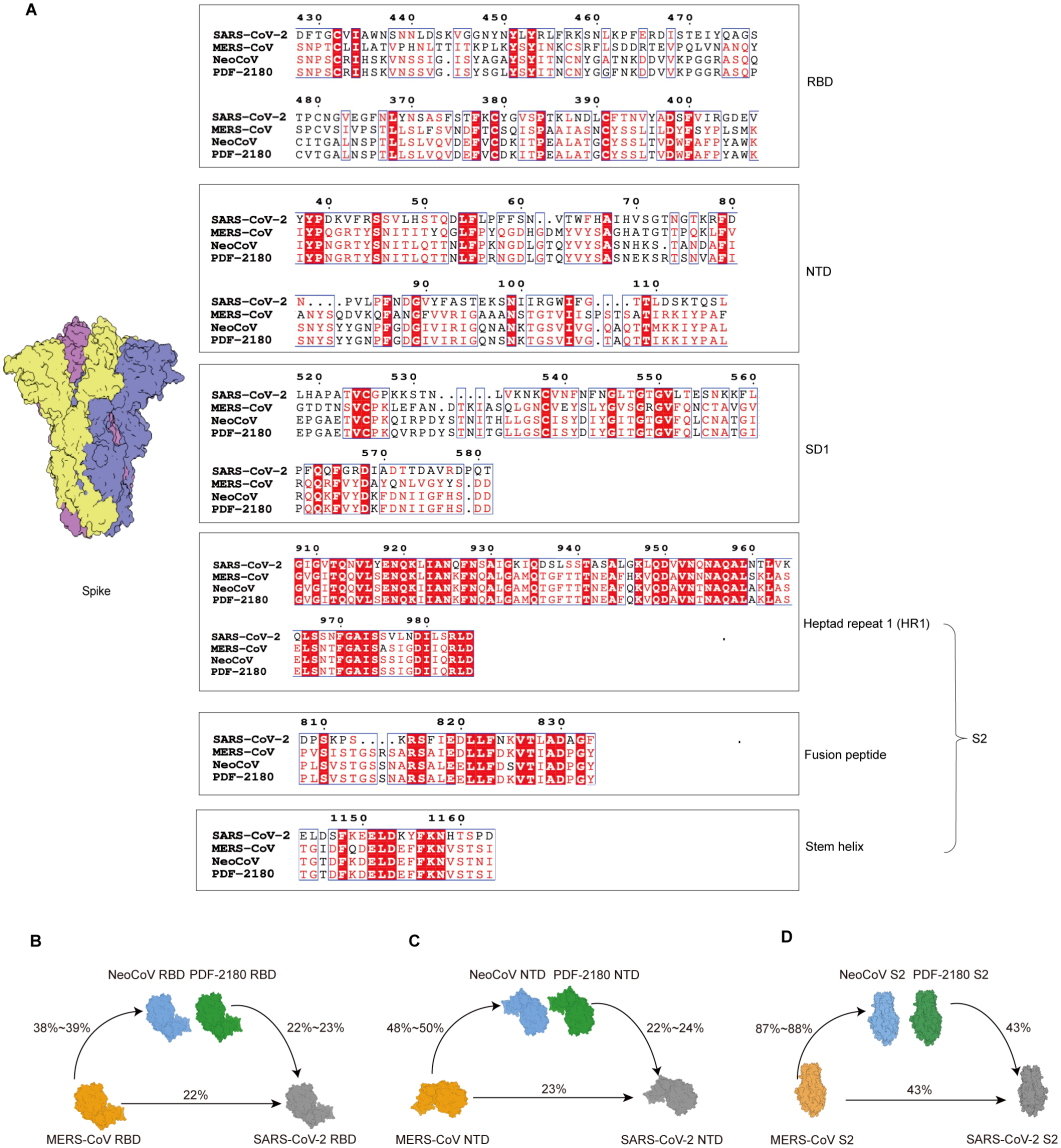
Sequence similarity analysis of S proteins of distinct β-coronaviruses. **(A)** Amino acid sequence alignment of distinct domains or motifs on S proteins. The identical amino acid residues among the four β-coronaviruses are labeled in red. **(B-D)** Amino acid sequence similarities of RBD **(B)**, NTD **(C)**, and S2 **(D)** domains among wildtype SARS-CoV-2, MERS-CoV and its related coronaviruses, NeoCoV and PDF-2180.

We then performed amino acid sequence alignment of the S proteins from our interested four coronaviruses, SARS-CoV-2, MERS-CoV, NeoCoV, and PDF-2180 (**Supplementary Figure S3**). As shown, the sequence similarities of the entire S1 domain, including the RBD, NTD, and SD1 regions, were very low (**Figure 4A**, **Supplementary Figure S3**), with only 22~24% amino acids shared between SARS-CoV-2 and MERS/MERS-related coronaviruses (**Figures 4B, C**). These results explained why there was barely ELISA cross-reactivity for antibodies against RBD and NTD. For example, most of the amino acid residues for the binding of antibody sd1.040 on SARS-CoV-2 SD1 (14) were radically different from the SD1 sequences of MERS/MERS-related coronaviruses (**Supplementary Figure S2D**).

Nevertheless, the sequence alignment suggested a higher level of similarity of S2 region (**Supplementary Figure S3**), with 43% of amino acid similarity between S2 proteins of SARS-CoV-2 and MERS/MERS-related coronaviruses (**Figure 4D**). Especially, several conserved segments on S2 with highly conserved amino acid residues were identified, such as fusion peptide and stem helix regions (**Figure 4A**).

### Cross-reactivity of anti-S2 antibodies

**Figure 5:**
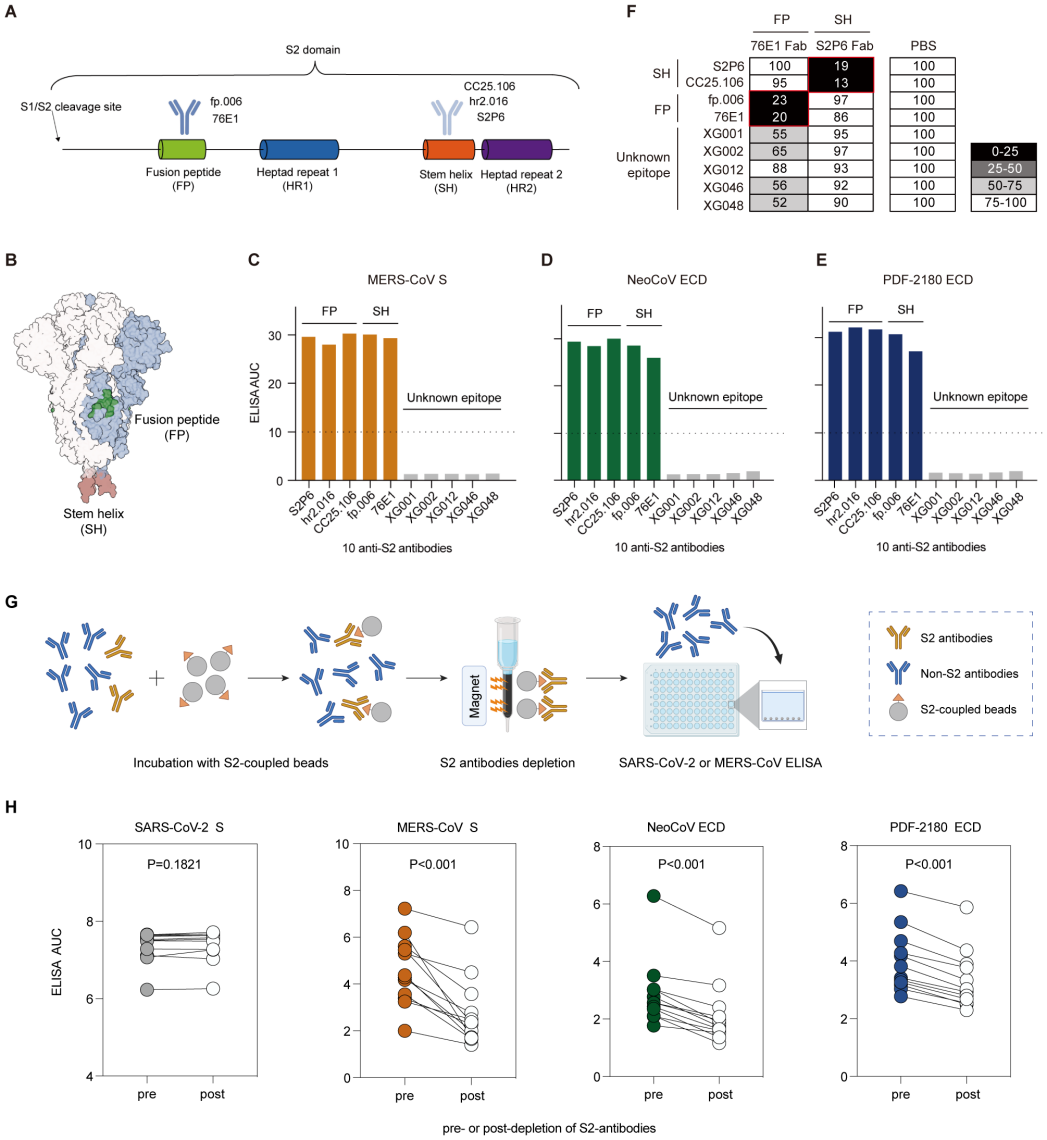
Antibody cross-reactivity for anti-S2 antibodies. **(A)** Schematic diagram of the S2 domain and S2-binding antibodies. Antibodies recognizing the fusion peptide (FP), fp.006 (14) and 76E1 (38); and antibodies recognizing the stem helix (SH) region, CC25.106 (37), S2P6 (36), and hr2.106 (14). **(B)** Structure of the SARS-CoV-2 S trimer. Two conserved epitopes, fusion peptide (green) and stem helix region (red), on the S2 domain are labeled. **(C–E)** ELISA binding activity of several S2-binding antibodies against the S proteins of MERS-CoV **(C)**, NeoCoV **(D)**, and PDF-2180 **(E)**. Representative of at least two independent experiments. **(F)** Competition ELISA among S2-targeting antibodies. The antibodies in the x-axis are 1st antibodies (Fab segments) for epitope blocking. The antibodies in the y-axis are 2nd antibodies (IgG1) for signal detection. Samples without 1st antibody blocking (PBS buffer substituted for the 1st antibody blocking) were used as a reference for normalization. The percentage of relative binding: black, 0%–25%; dark gray, 25%–50%; light gray, 50%–75%; and white, >75%. All of the tested antibodies blocked the binding of their own Fabs efficiently. The red box indicates different epitopes. **(G)** Depletion of S2 binding antibodies from human serum samples. **(H)** Binding of serum samples against the S proteins of SARS-CoV-2, MERS-CoV, NeoCoV and PDF-2180 before and after depletion of S2 binding antibodies measured by ELISA area under the curve (AUC). Statistical significance differences are determined by paired t-test.

Based on the homology analysis, we hypothesized that the serum cross-reactivity against S proteins of MERS/MERS-related coronaviruses might be elicited by cross-reactive anti-S2 antibodies. To test this, we then focused on two relatively conserved epitopes on S2, the fusion peptide (aa 816-834) and stem helix (aa 1139-1163) regions on S2 domain (**Figures 5A, B**), both of which were folded as α-helical conformations, extending an amphipathic helix in S trimer structure (35).

We selected 10 monoclonal antibodies, including S2P6 (36), CC25.106 (37), and hr2.016 (14) targeting the stem helix region, fp.006 (14) and 76E1 (38) targeting the fusion peptide, and five S2-binding XG antibodies isolated from a convalescent donor (5). We evaluated their ELISA cross-reactivity and showed that five of them (S2P6, CC25.106, hr2.016, fp.006, and 76E1) maintained their potent binding activity against the S proteins of MERS-CoV, NeoCoV and PDF-2180 (**Figures 5C–E**). However, five S2-binding XG antibodies we cloned from a convalescent donor showed no cross-binding activity, possibly due to that their uncharacterized S2 epitopes were not conserved. We then performed competition ELISA among these 10 antibodies (**Figure 5F**), and the results showed that the five S2 binding XG antibodies did not compete with those targeting the SH and FP regions, suggesting that they recognize distinct epitopes.

To further investigate the role of S2-binding antibodies in cross-reactivity, we examined serum samples from BA.5/BF.7 breakthrough infections. To test this, we depleted serum samples of antibodies that bind to the S2 domain of SARS-CoV-2, or performed a mock depletion, and measured the residual binding activity against S proteins of SARS-CoV-2, MERS-CoV, NeoCoV and PDF-2180 (**Figure 5G**). We found that for all 12 tested serum samples, depletion of SARS-CoV-2 S2-binding antibodies statistically significantly reduced the binding activities against MERS-CoV, NeoCoV and PDF-2180 (**Figure 5H**). However, the depletion exhibited no significant effect on the binding to the S protein of SARS-CoV-2 (**Figure 5H**). This discrepancy was probably due to the fact that among the total antibody pool induced by infection, S2-specific antibodies only constitute a small proportion. Consequently, the remaining RBD- or NTD-binding antibodies exhibited strong ELISA reactivity against the S protein of SARS-CoV-2, but not of MERS-CoV, NeoCoV and PDF-2180. Therefore, these results strengthen our conclusion that the S2-binding antibodies are responsible for the cross-reactivity against MERS and MERS-like coronaviruses.

Taken together, among the serum samples and monoclonals we tested, only anti-S2 antibodies against SARS-CoV-2 exhibited strong cross-reactivity against the S proteins of MERS-CoV, and its two related merbecoviruses, NeoCoV and PDF-2180. Combined with the S2 sequence similarity between SARS-CoV-2 and MERS/MERS-related coronaviruses, we concluded that the serological cross-reactivity against the S proteins of MERS/MERS-related coronaviruses was probably due to the anti-S2 antibodies induced by SARS-CoV-2 infection.

### *In vitro* cross-neutralizing activity against MERS/MERS-related coronaviruses

**Figure 6:**
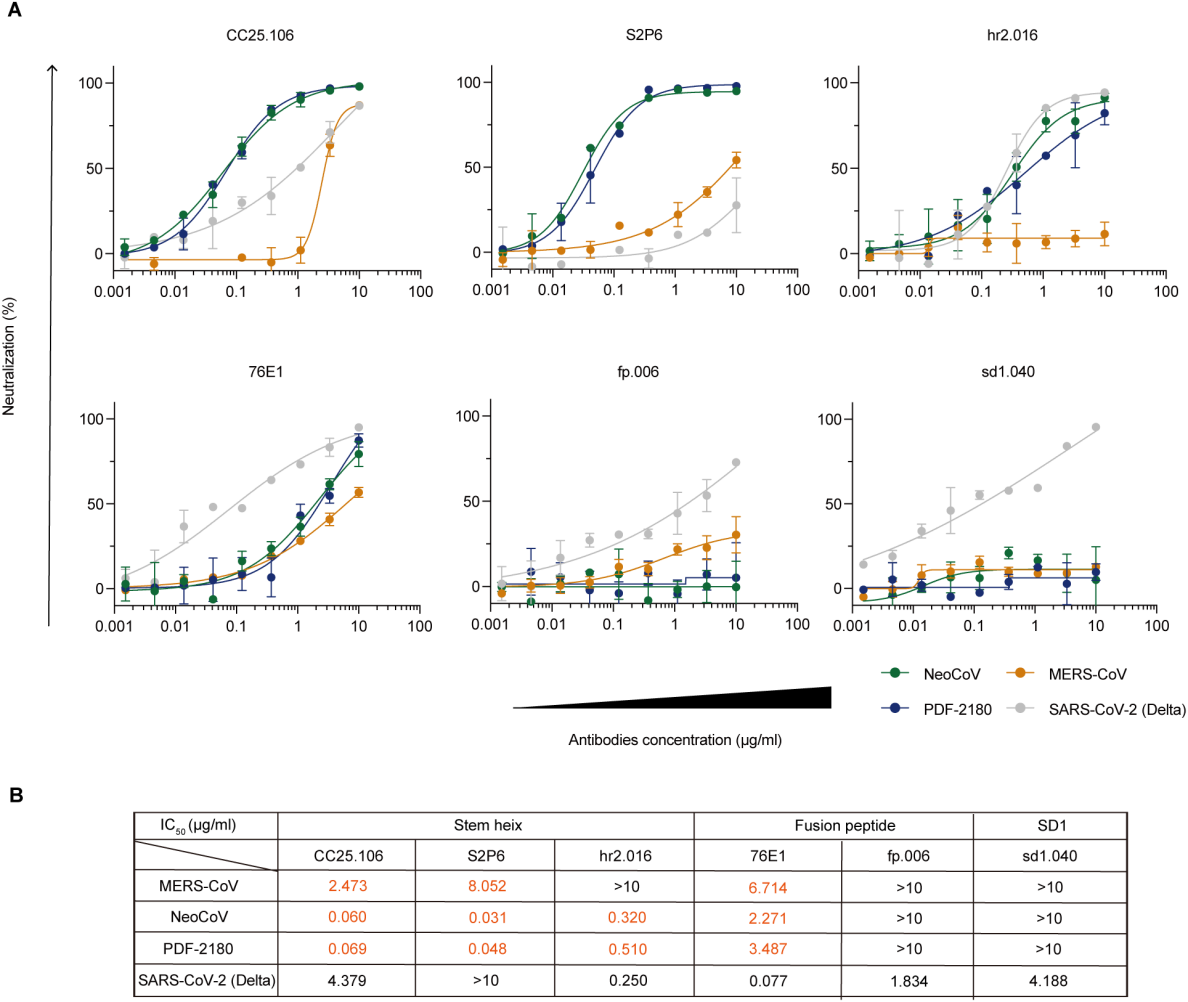
*In vitro* neutralization against MERS/MERS-related coronaviruses. **(A)** *In vitro* neutralization assays using pseudoviruses against SARS-CoV-2 Delta (gray), MERS-CoV (orange), NeoCoV (green), and PDF-2180 (blue). Percent neutralization of infection in the presence of the indicated antibodies CC25.106, S2P6, hr2.016, 76E1, fp.006 and sd1.040 is shown normalized to infection without antibody addition. Representative of at least two independent experiments. Data are shown as mean ± SD. **(B)** Calculated IC_50_ values.

To further study the biological function of these anti-S2 cross-reactive antibodies and to examine whether they could cross-neutralize MERS/MERS-related coronaviruses, we performed *in vitro* neutralization assays by using luciferase-expressing pseudoviruses to infect Huh-7 cells (for SARS-CoV-2 Delta and MERS-CoV pseudoviruses) and HEK293T-mutACE2 cells (for NeoCoV and PDF-2180 pseudoviruses) (See Methods). Among the five tested anti-S2 monoclonal antibodies, four (CC25.106, S2P6, hr2.016, and 76E1) showed cross-neutralizing activity against NeoCoV and PDF-2180 pseudoviruses (**Figure 6A**), with IC_50_ values ranging from 0.031 to 3.487 μg/ml (**Figure 6B**). As a negative control, the SD1-binding but no cross-reactive antibody, sd1.040, showed no cross-neutralizing activity (**Figures 6A, B**).

Specifically, antibodies against stem helix (CC25.106) and fusion peptide (76E1) showed neutralizing capacity against all four tested viruses, including SARS-CoV-2 Delta and MERS/MERS-related coronaviruses (**Figure 6B**). Interestingly, both anti-stem helix antibodies, CC25.106 and S2P6, showed more potent neutralizing activity against NeoCoV (IC_50_: 0.060 μg/ml for CC25.106; 0.031 μg/ml for S2P6) and PDF-2180 (IC_50_: 0.069 μg/ml for CC25.106; 0.048 μg/ml for S2P6) than against MERS-CoV and SARS-CoV-2 Delta (**Figure 6B**). Crystal structure analysis reveals that S2P6 (36) (PDB: 7RNJ) and CC25.106 (37) (PDB: 8DGU) have highly similar epitopes on the stem helix peptide of SARS-CoV-2 spike (**Supplementary Figure S4A**). Sequence alignment indicates that the stem helix of NeoCoV and PDF-2180 are highly conserved, but with differences from SARS-CoV-2, including Y1155 (**Supplementary Figure S4A**). In NeoCoV and PDF-2180, the substitution of tyrosine for phenylalanine, although it diminishes hydrogen bond interactions, may correlate with the formation of a larger and more stable hydrophobic pocket due to interactions with surrounding hydrophobic residues. The Y1155F mutant did not significantly impair the binding of S2P6 to the SARS-CoV-2 stem helix, thereby indirectly supporting this hypothesis (36). Furthermore, S2P6 exhibits interactions with S1147, where the replacement of serine with aspartic acid in NeoCoV and PDF-2180 likely introduces additional hydrogen bonding interactions, thereby enhancing the stability of their association (**Supplementary Figure S4B**).

On the contrary, the antibody hr2.016 recognizing also stem helix neutralized NeoCoV (IC_50_: 0.320 μg/ml) and PDF-2180 (IC_50_: 0.510 μg/mL), similar to SARS-CoV-2 Delta (IC_50_: 0.250 μg/ml), but lost its neutralizing capacity against MERS-CoV (IC_50_: >10 μg/ml), implying the conformational similarity among hr2.016 epitopes on SARS-CoV-2, NeoCoV, and PDF-2180, but not on MERS-CoV.

### *In vitro* antibody-dependent enhancement effect of viral entry

Anti-SARS-CoV-2 S protein antibodies could be a double-edged sword. During their protection against viral entry, some antibodies might utilize their binding capacity with Fc receptor and facilitate the viral entry into the FcR-positive cells, leading to an enhancement of viral entry and even the illness (39, 40).

**Figure 7:**
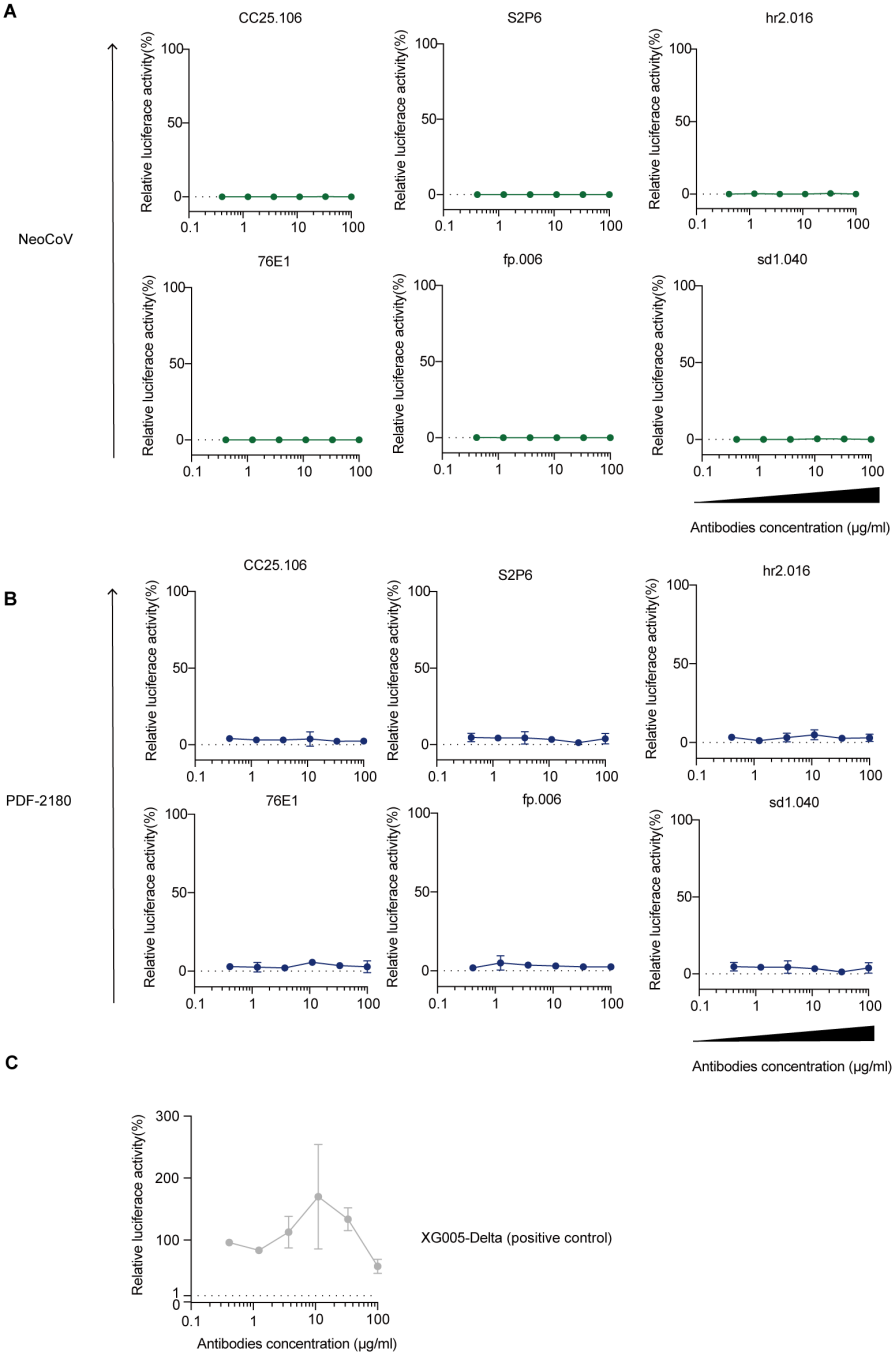
Raji cell-dependent *in vitro* ADE analysis. **(A-C)** ADE assay against NeoCoV **(A)** and PDF-2180 **(B)**. XG005 (47) as a positive antibody control for ADE of viral entry **(C)**. Representative of at least two independent experiments. Data are presented as the mean ± SD.

To investigate whether these cross-reactive monoclonal antibodies could induce ADE of viral entry for MERS/MERS-related coronaviruses, we used the luciferase-expressing NeoCoV and PDF-2180 pseudoviruses and established Raji cell-dependent system (5) to study the *in vitro* ADE effect of NeoCoV/PDF-2180 entry. As shown, none of our tested anti-S2 cross-reactive antibodies induced ADE of viral entry (**Figures 7A, B**); while XG005, as a positive control, induced robust luciferase activity, which was an indicator of entry of SARS-CoV-2 Delta pseudoviruses (**Figure 7C**). Together, in our assay, no ADE of viral entry against NeoCoV and PDF-2180 was detected for the cross-reactive anti-S2 antibodies.

## Discussion

Both sarbecovirus, such as SARS-CoV-2 and its variants, and merbecovirus, such as MERS-CoV, pose a great threat to the global health (9). The ACE2 receptor-using MERS-related coronaviruses, NeoCoV and PDF-2180, and their further evolution (10), might lead to a potential recombination between SARS-CoV-2 and MERS-related coronaviruses, raising the risk of a newly emerging β-CoV clade, such as SARS-CoV-3 or MERS-CoV-2, for another catastrophic epidemic (12).

In this study, we found that some donors with SARS-CoV-2 breakthrough infection, but not donors receiving inactivated SARS-CoV-2 vaccines, have serological ELISA cross-reactivity against MERS-CoV and its relatives, NeoCoV and PDF-2180. This cross-reactivity was probably due to the presence of anti-S2 antibodies recognizing the S2 conversed epitopes, including but not limited to the fusion peptide and stem helix regions. Moreover, these cross-reactive anti-S2 antibodies might also exhibit cross-neutralizing activity against MERS/MERS-related coronaviruses, but induced no ADE of viral entry.

Interestingly, the serological cross-reactivity against MERS/MERS-related coronaviruses was only induced by SARS-CoV-2 breakthrough infection, but not by inactivated SARS-CoV-2 vaccination. This observation could be explained by the fact that SARS-CoV-2 infection induced many anti-S2 antibodies, even more than antibodies targeting other epitopes, such as RBD and NTD (41). On the other hand, vaccination with SARS-CoV-2 inactivated vaccines induced only a few anti-S2 antibodies (19, 42). Therefore, the anti-S2 antibodies cross-reactive with MERS-CoV and its relative coronaviruses are more likely to be induced by SARS-CoV-2 infection but not by inactivated SARS-CoV-2 vaccination.

Beyond the MERS/MERS-related coronaviruses we tested in this study, it is highly likely that these cross-reactive anti-S2 antibodies also cross-reacts with other β-coronaviruses, including but not limited to MERS-CoV, NeoCoV and PDF-2180. Therefore, based on this study, we speculated that to develop an efficient pan-β-CoV vaccine for protection against both merbecoviruses and sarbecoviruses, the relative conserved S2 domain would be a potential antigen candidate, so that the anti-S2 cross-reactive antibodies against all β-coronaviruses could be induced.

## Materials and methods

### Collection of human serum samples

Volunteer recruitment and blood draws were performed at Fudan University in Shanghai under a protocol approved by the institutional Ethics Committee (2022-C005). The recruited volunteers included 20 unexposed (unvaccinated and uninfected) donors, 21 vaccinated donors who had received two or three doses of inactivated vaccines but had never been infected by SARS-CoV-2, and 21 BA.5/BF.7 breakthrough infected individuals during the COVID-19 epidemic in mainland China between November 2022 and January 2023 (43). Experiments related to all human samples were performed at the School of Basic Medical Sciences, Fudan University.

### Expression and purification of monoclonal antibodies

Antibody cloning and expression were performed as previously reported (5, 44). Briefly, the plasmids encoding the antibody heavy/light chains were co-transfected into human embryonic kidney 293F (HEK293F) cells maintained in serum-free OPM-293-CD05 medium (OPM Biosciences) in a shaking incubator at 100 rpm, 37°C, 5% CO_2_. The supernatant was harvested after a seven-day culture and incubated with protein G agarose (GenScript) for affinity purification following the manufacturer’s instructions. The eluted antibodies were collected, concentrated by ultrafiltration and stored for the following analysis.

### Expression and purification of viral proteins

The S protein of MERS-CoV (GenBank: YP_009047204.1) was cloned into the pcDNA3.1 vector with a C-terminal T4 DNA fibrin and eight-histidine tag (His-tag). The ectodomains (ECD) of NeoCoV (GenBank: AGY29650.2) and PDF-2180 (GenBank: YP_009361857.1) were cloned into the pCAGGS vector, with a C-terminal His-tag added for protein purification. Moreover, MERS-CoV RBD (aa 367-588), MERS-CoV NTD (aa 18-353), NeoCoV RBD (aa 380-585), NeoCoV NTD (aa 20-353), PDF-2180 RBD (aa 381-586), PDF-2180 NTD (aa 20-336) were cloned similarly with a C-terminal His-tag added. All these plasmids were transfected into serum-free OPM-293-CD05 medium-cultured HEK293F cells using transfection reagent EZ Trans (Life-iLab Biotech), respectively. After culture for five days, the supernatant was harvested and incubated with Ni Sepharose excel (Cytiva) at 4˚C overnight for affinity purification. After wash with 20 mM imidazole, the bound His-tagged proteins were eluted with 500 mM imidazole-containing elution buffer to collect a total of 20 ml of eluate. The eluted protein was concentrated by ultrafiltration, aliquoted, and stored for ELISA.

### Depletion of S2 antibodies from serum samples

Streptavidin-Magnetic beads (Acro Biosystems) were prepared according to the manufacturer’s instructions. Briefly, beads were washed three times with assay buffer (PBS with 0.05% w/v BSA) and incubated with biotinylated S2 protein of SARS-CoV-2 at 50 μg per mg beads for one hour at room temperature in a rotary mixer. Beads were then washed four times and reconstituted at 1 mg/ml in assay buffer. Beads were incubated with human serum sample at a 3:1 ratio of beads: plasma, rotating overnight at 4°C. After incubation, tubes were placed on a magnetic holder and supernatants were collected for the following assays.

### Enzyme-linked immunosorbent assay

ELISA was performed as previously described (5). Briefly, 96-well plates were coated with antigens (5 μg/ml or 10 μg/ml) diluted in phosphate-buffered saline (PBS) overnight at 4˚C. Antigen proteins included S protein ectodomain (S-ECD), RBD, NTD proteins of MERS-CoV, NeoCoV, PDF-2180 (5 μg/ml) and SARS-CoV-2 (10 μg/ml). After wash with 0.05% Tween-20 in PBS (PBST), the plates were then blocked with PBS containing 3% bovine serum albumin (BSA), and incubated with the 1st antibody (3-fold dilution, 8 gradients with a maximum concentration of 10 μg/ml) in PBS for 1 hour at room temperature. After wash with PBST, the 2nd HRP-conjugated goat anti-human IgG antibody (Thermo Fisher Scientific) was added for incubation, and then subjected to luminescence measurement using a plate-based luminometer. The area under the curve (AUC) values were then calculated for each serum sample and monoclonal antibody to evaluate the antigen-binding capacity using PRISM software. Samples with AUC value >10 were considered reactive, indicating positive binding.

### Competition ELISA

96-well plates were coated with 1 μg/ml SARS-CoV-2 S protein diluted in phosphate-buffered saline (PBS) overnight at 4°C and then blocked with PBS containing 3% bovine serum albumin (BSA) for 2 hours. After blocking, plates were pre-incubated with 500 μg/ml 1st blocking antibody (Fab segments without constant region of IgG1) for 2 hours at room temperature. And then, 1 μg/ml of 2nd antibodies were directly added and incubated for 30 minutes (full length IgG1). Detection was performed using HRP-conjugated goat anti-human IgG Fc antibody (Thermo Fisher Scientific). PBS buffer substituted for the first blocking antibody was used as a reference for normalization.

### Generation of pseudoviruses

Human embryonic kidney 293T (HEK293T) cells were cultured in Dulbecco’s Modified Eagle Medium (DMEM) supplemented with 10% heat-inactivated fetal bovine serum (FBS). The pseudotyped viruses of MERS-CoV, NeoCoV and PDF-2180 were generated as previously described (45). Briefly, the S protein sequences of MERS-CoV, NeoCoV and PDF-2180 were cloned into the pcDNA3.1 vector. The constructed pcDNA3.1-MERS-CoV-S, pcDNA3.1-NeoCoV-S, and pcDNA3.1-PDF-2180-S plasmids were then co-transfected with pNL4-3.luc.RE (the luciferase reporter-expressing HIV-1 backbone) into HEK293T cell lines by using the transfection reagent VigoFect (Vigorous Biotech). Six hours post-transfection, the cultured medium was replaced with fresh DMEM containing 10% FBS. Two days later, the supernatant containing pseudoviruses were centrifugated, collected, and stored at -80°C for *in vitro* neutralization assays.

### Pseudotyped virus-based *in vitro* neutralization assay

To perform pseudotyped virus-based *in vitro* neutralization assay, we used Huh‐7 cell lines for MERS-CoV and SARS-CoV-2 Delta pseudoviral infection and used HEK293T-mutACE2 cell lines for pseudoviral infection against NeoCoV and PDF-2180. Human hepatoma Huh-7 cells were cultured in DMEM supplemented with 10% heat-inactivated FBS. To generate the HEK293T-mutACE2 cell lines, we synthesized a type of chimeric human ACE2 receptor containing six mutations from bat ACE2 receptor (Bat37) (10) and cloned it into pMX-empty-IRES-EGFP vector. The constructed pMX-mutACE2-IRES-EGFP was then transfected into HEK293T cells, and the expression of EGFP was used to determine the transfection rate. The successfully transfected HEK293T-mutACE2 cells were used for the subsequent neutralization experiments against NeoCoV and PDF-2180 pseudoviruses. For *in vitro* neutralization assays, we first verified the pseudoviral infection through measuring the luciferase activity, and then we performed the *in vitro* neutralization assays in the presence of monoclonals (threefold serially diluted, nine dilutions with maximum concentration of 10 µg/ml). After 36-hour incubation and culture, the cells were lysed and subjected to luciferase activity measurement by using Firefly Luciferase Assay Kit (Promega) and a spectrophotometer (Tecan Infinite M200Pro) following the manufacturer’s instructions. To calculate the relative neutralizing activity, the absolute luciferase activity values from all wells were normalized to the luciferase values of virus-only control wells. The half-maximal inhibitory concentrations (IC_50_) values were then calculated by using nonlinear regression analysis in PRISM software.

### *In vitro* ADE of viral entry assay

The pseudovirus-based *in vitro* ADE assay was performed as previously described (5, 46). Briefly, the Raji cells, originated from human Burkitt’s lymphoma, were maintained in RPMI 1640 supplemented with 10% FBS at 37°C in 5% CO_2_, and were seeded into 96-well plates coated with polylysine. After 24 hours, the pseudovirus and antibody were mixed and then added into cultured cells. The tested monoclonal antibodies were serially diluted 1:3 with six dilutions in total (maximal concentration: 100 μg/ml). After 60-hour incubation, the Raji cells were lysed and the luciferase activity was measured by using Firefly Luciferase Assay Kit (Promega) a spectrophotometer (Tecan Infinite M200Pro).

### Statistical analysis

The ELISA AUC value and the neutralization titer IC_50_ were calculated in PRISM. Statistical significance differences are determined using one-way ANOVA or paired t-test. The detailed calculation methods for statistical analysis are indicated in the Result part or figure legends.

## Data Availability

The original contributions presented in this study are included in the article/**Supplementary Material.**

## Supplementary material

Supplementary Figure 1 Protein expression and purification. **(A)** Schematic diagram of SARS-CoV-2 S protein. **(B)** Protein electrophoresis of mammalian cell-expressed recombinant S, RBD, and NTD proteins of MERS-CoV, NeoCoV, and PDF-2180.

Supplementary Figure 2 Antibody ELISA for S1- but not RBD/NTD-binding antibodies. **(A-C)** Antibody ELISA binding against S protein MERS-CoV **(A)**, NeoCoV **(B)**, and PDF-2180 **(C)**. An S2-binding antibody S2P6 as a positive control. At least two independent experiments. **(D)** Amino acid sequence alignment. Blue triangles indicate the binding sites of the SD1-binding antibody sd1.040 on the S protein of SARS-CoV-2 (PDB: 8D48).

Supplementary Figure 3 S protein amino acid sequence alignment. Amino acid sequences of S proteins for SARS-CoV-2, MERS-CoV, NeoCoV, and PDF-2180 are aligned. Identical amino acid residues are labeled in red. The orange line represents the S2 domain (aa 706-1211). The fusion peptide (FP) and stem helix (SH), two conserved motifs within S2 domain, are marked by green and purple rectangles, respectively.

Supplementary Figure 4 Structural analysis of S2P6 and CC25.106 binding to the stem helix of SARS-CoV-2, MERS-CoV and MERS-related coronaviruses. **(A)** S stem helix sequence alignment of SARS-CoV-2, MERS-CoV, NeoCoV and PDF-2180. Conserved amino acids are highlighted in red. Residues involved in S2P6 binding are marked in orange, and those involved in CC25.106 binding are marked in blue. **(B)** Detailed interactions between SARS-CoV-2 S stem helix Y1155 and antibody S2P6 (PDB: 7RNJ) and antibody CC25.106 (PDB: 8DGU). Hydrogen bonds are indicated as dotted lines.
