# Supplementary figures and images for: Anti-S2 antibodies responsible for the SARS-CoV-2 infection-induced serological cross-reactivity against MERS-CoV and MERS-related coronaviruses
Source: Front Immunol. 2025 Mar 28;16:1541269. doi: 10.3389/fimmu.2025.1541269 (PMC11985752; doi:10.3389/fimmu.2025.1541269)

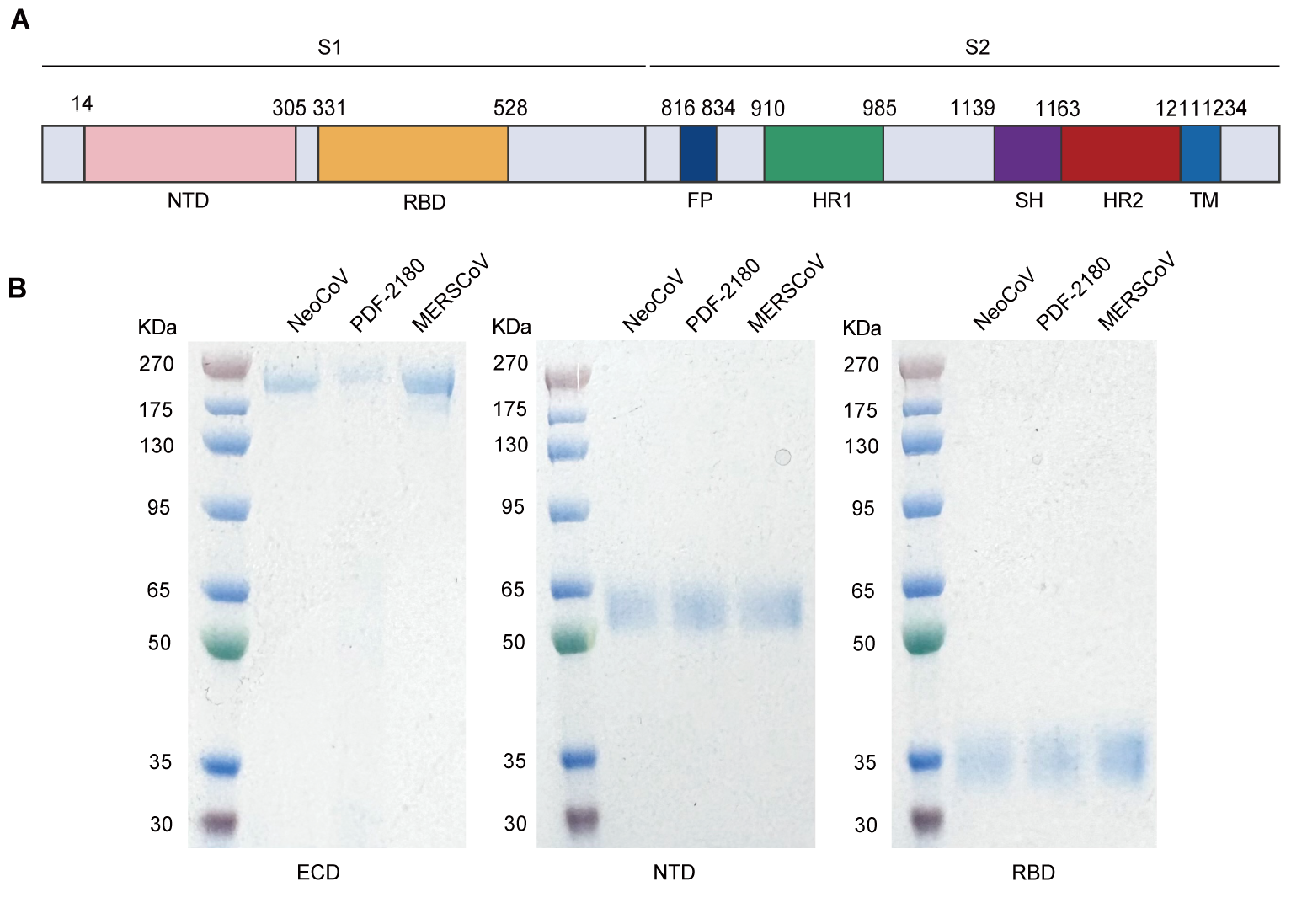

Supplement: Supplementary Figure 1 — Protein expression and purification. (A) Schematic diagram of SARS-CoV-2 S protein. (B) Protein electrophoresis of mammalian cell-expressed recombinant S, RBD, and NTD proteins of MERS-CoV, NeoCoV, and PDF-2180. [file Image1.tif]

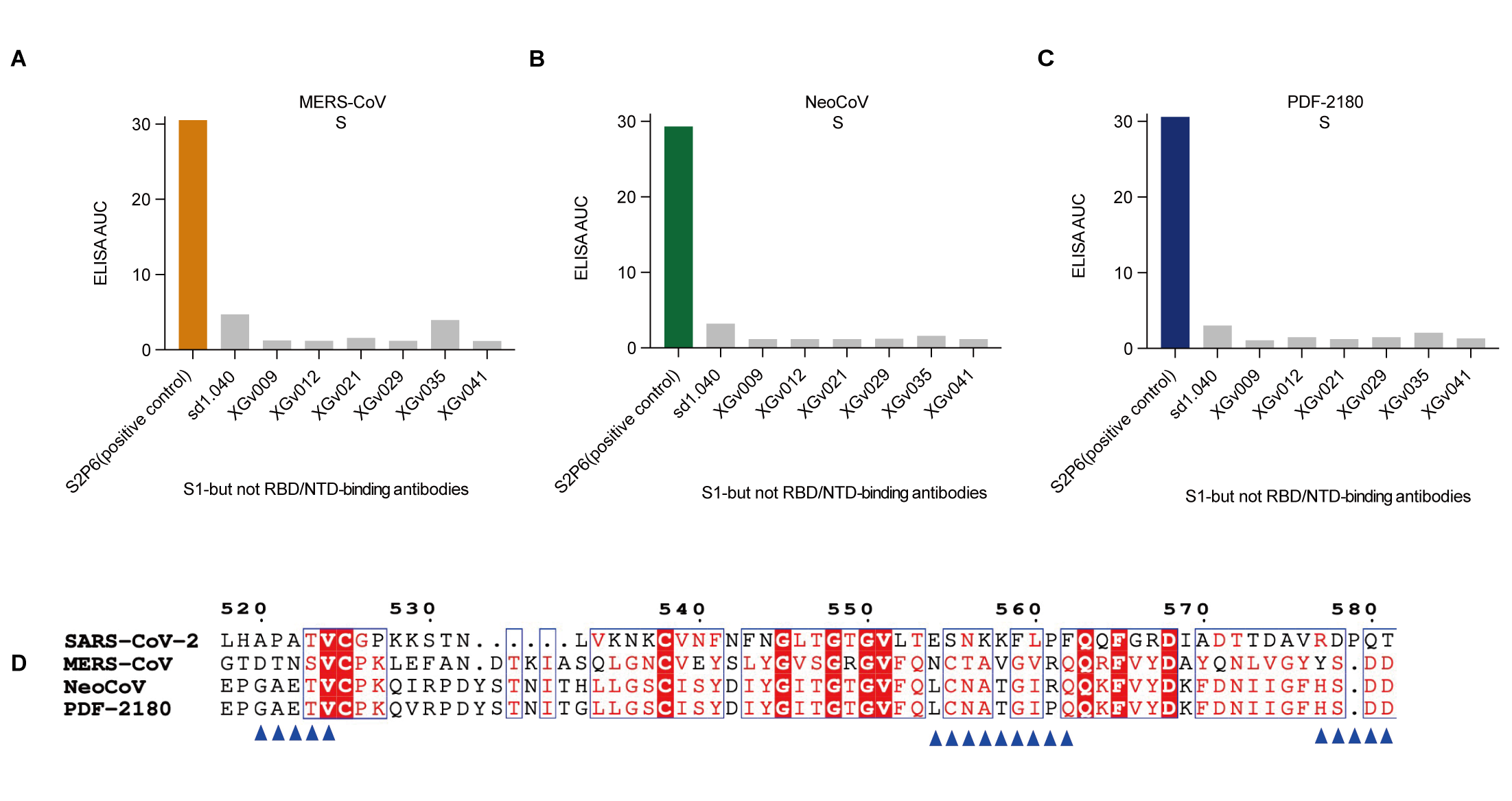

Supplement: Supplementary Figure 2 — Antibody ELISA for S1- but not RBD/NTD-binding antibodies. (A-C) Antibody ELISA binding against S protein MERS-CoV (A), NeoCoV (B), and PDF-2180 (C). An S2-binding antibody S2P6 as a positive control. At least two independent experiments. (D) Amino acid sequence alignment. Blue triangles indicate the binding sites of the SD1-binding antibody sd1.040 on the S protein of SARS-CoV-2 (PDB: 8D48). [file Image2.tif]

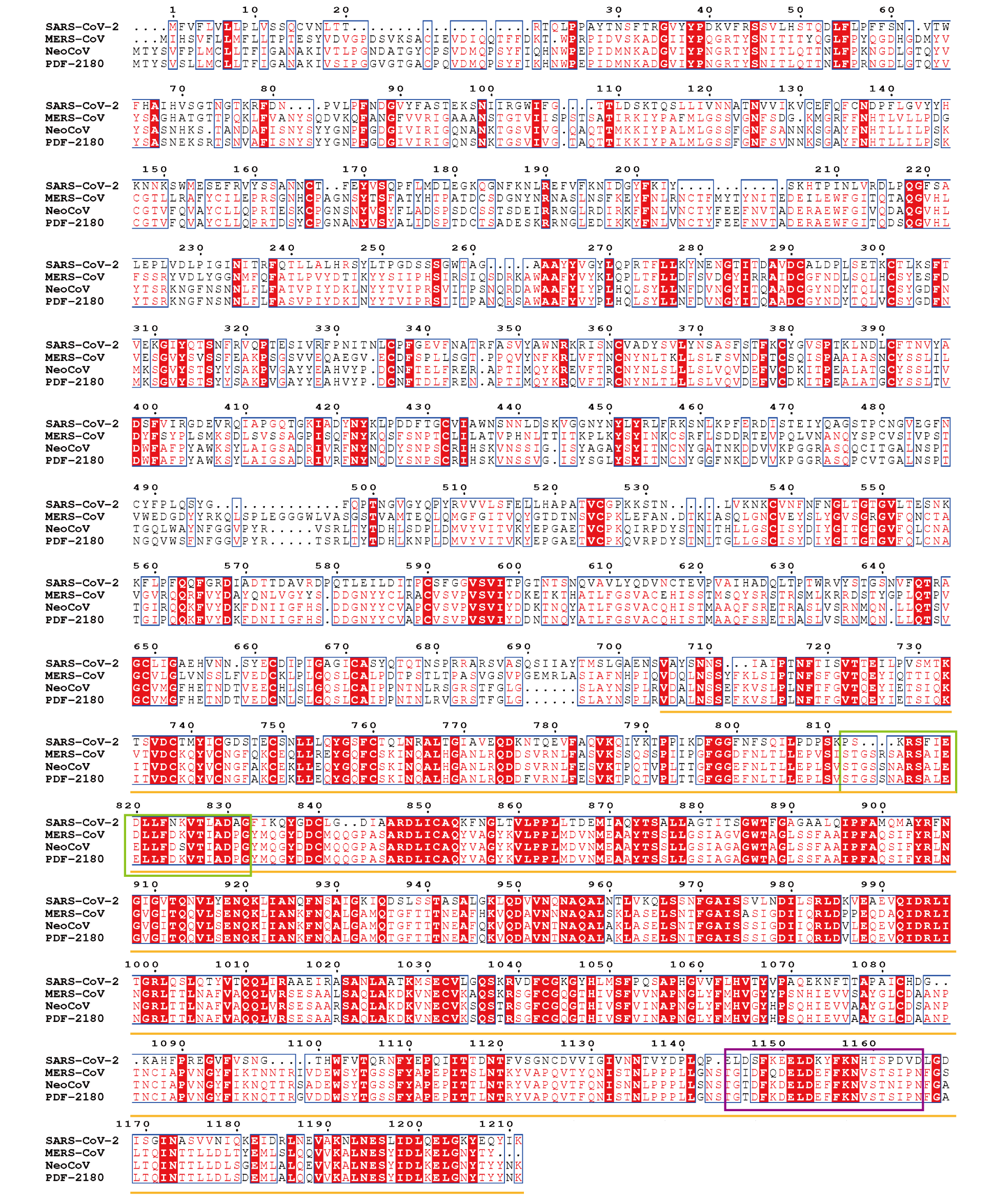

Supplement: Supplementary Figure 3 — S protein amino acid sequence alignment. Amino acid sequences of S proteins for SARS-CoV-2, MERS-CoV, NeoCoV, and PDF-2180 are aligned. Identical amino acid residues are labeled in red. The orange line represents the S2 domain (aa 706-1211). The fusion peptide (FP) and stem helix (SH), two conserved motifs within S2 domain, are marked by green and purple rectangles, respectively. [file Image3.tif]

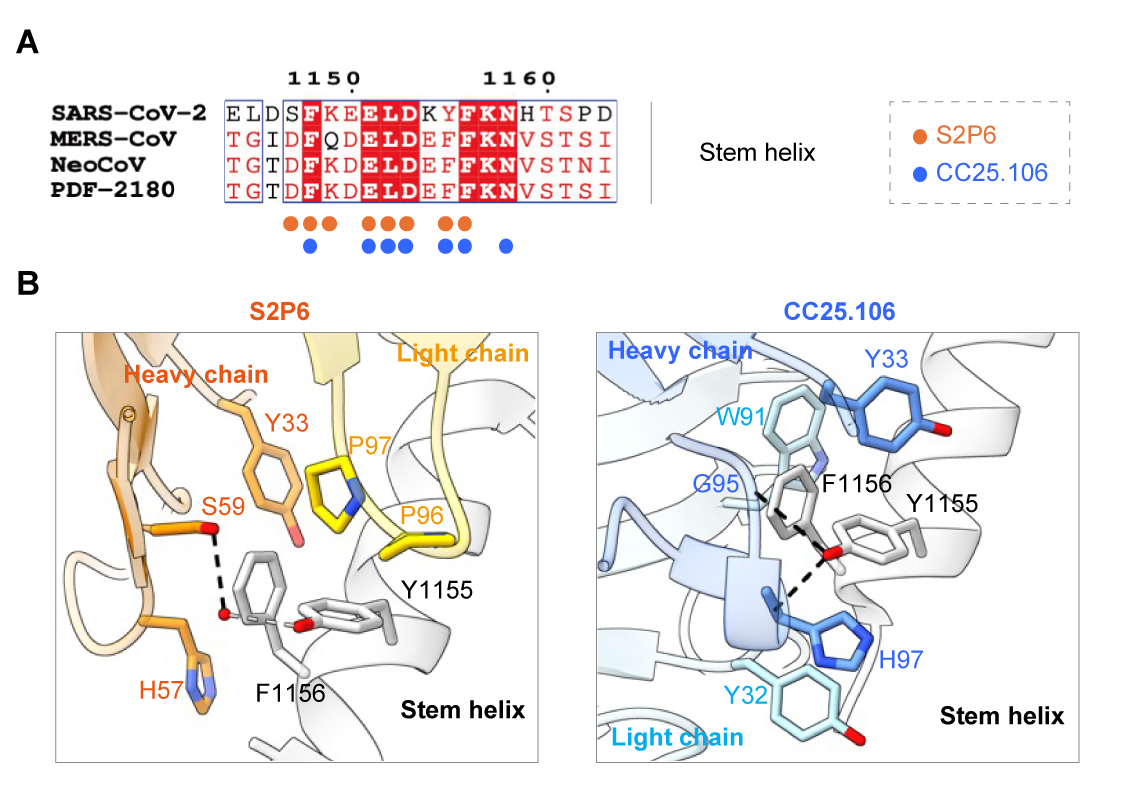

Supplement: Supplementary Figure 4 — Structural analysis of S2P6 and CC25.106 binding to the stem helix of SARS-CoV-2, MERS-CoV and MERS-related coronaviruses. (A) S stem helix sequence alignment of SARS-CoV-2, MERS-CoV, NeoCoV and PDF-2180. Conserved amino acids are highlighted in red. Residues involved in S2P6 binding are marked in orange, and those involved in CC25.106 binding are marked in blue. (B) Detailed interactions between SARS-CoV-2 S stem helix Y1155 and antibody S2P6 (PDB: 7RNJ) and antibody CC25.106 (PDB: 8DGU). Hydrogen bonds are indicated as dotted lines. [file Image4.tif]
